# HIV Screening via Fourth-Generation Immunoassay or Nucleic Acid Amplification Test in the United States: A Cost-Effectiveness Analysis

**DOI:** 10.1371/journal.pone.0027625

**Published:** 2011-11-16

**Authors:** Elisa F. Long

**Affiliations:** School of Management, Yale University, New Haven, Connecticut, United States of America; Massey University, New Zealand

## Abstract

**Background:**

At least 10% of the 56,000 annual new HIV infections in the United States are caused by individuals with acute HIV infection (AHI). It unknown whether the health benefits and costs of routine nucleic acid amplification testing (NAAT) are justified, given the availability of newer fourth-generation immunoassay tests.

**Methods:**

Using a dynamic HIV transmission model instantiated with U.S. epidemiologic, demographic, and behavioral data, I estimated the number of acute infections identified, HIV infections prevented, quality-adjusted life years (QALYs) gained, and the cost-effectiveness of alternative screening strategies. I varied the target population (everyone aged 15-64, injection drug users [IDUs] and men who have sex with men [MSM], or MSM only), screening frequency (annually, or every six months), and test(s) utilized (fourth-generation immunoassay only, or immunoassay followed by pooled NAAT).

**Results:**

Annual immunoassay testing of MSM reduces incidence by 9.5% and costs <$10,000 per QALY gained. Adding pooled NAAT identifies 410 AHI per year, prevents 9.6% of new cases, costs $92,000 per QALY gained, and remains <$100,000 per QALY gained in settings where undiagnosed HIV prevalence exceeds 4%. Screening IDUs and MSM annually with fourth-generation immunoassay reduces incidence by 13% with cost-effectiveness <$10,000 per QALY gained. Increasing the screening frequency to every six months reduces incidence by 11% (MSM only) or 16% (MSM and IDUs) and costs <$20,000 per QALY gained.

**Conclusions:**

Pooled NAAT testing every 12 months of MSM and IDUs in the United States prevents a modest number of infections, but may be cost-effective given sufficiently high HIV prevalence levels. However, testing via fourth-generation immunoassay every six months prevents a greater number of infections, is more economically efficient, and may obviate the benefits of acute HIV screening via NAAT.

## Introduction

Each year, more than 56,000 people in the United States acquire HIV, many of whom are infected by individuals with acute HIV infection (AHI), although the exact contribution of AHI is uncertain.[1]–[4] AHI typically lasts for two to three months after initial infection and individuals with AHI are exceptionally infectious during this period due to rapid viral replication,[2], [5], [6] because blood plasma viral loads are 100 times higher than during asymptomatic infection.[7] Moreover, individuals with AHI are likely status-unaware and may have had recent sexual contact with one or more partners.

Prior studies indicate that individuals identified with AHI may reduce risky sexual behavior.[8], [9] Successfully identifying such individuals during a short window may necessitate a frequent AHI screening program. Third-generation enzyme linked immunosorbent assays (ELISA) do not detect antibodies for at least three weeks after infection, and newer fourth-generation antigen-antibody combination tests reduce this window by several days. Before third-or fourth-generation assays detect infection, plasma viral RNA may be detected with a nucleic acid amplification test (NAAT). Individual NAAT screening is cost-prohibitive in many settings, and several studies have developed and piloted pooled NAAT testing, with the optimal pooling algorithm depending on undetected AHI prevalence.[6], [10]–[16] Pooled NAAT has been shown to be cost-effective in a community clinic serving high-risk men who have sex with men (MSM), although the study did not compare testing with a fourth-generation immunoassay.[17] Another study found that fourth-generation tests detect 62% of samples classified as acute infection, suggesting that newer immunoassays may obviate the need for NAAT testing.[18]

Recent guidelines recommend routine HIV screening of adults and adolescents aged 13 to 64,[19] but it is unknown to what extent concomitant efforts to increase AHI testing via NAAT will prevent new infections and whether such a strategy is cost-effective. Additionally, it is unclear whether NAAT testing should be utilized given that a fourth-generation immunoassay was approved by the U.S. Food and Drug Administration in June 2010.

Identifying the optimal HIV screening strategy, including which test(s) to administer, screening frequency, and target population, could potentially prevent thousands of new HIV infections, adding millions of life years to the population. The present study is the first to compare the population-level health benefits and costs of universal or targeted HIV screening with a fourth-generation immunoassay, versus screening for acute infection with pooled NAAT.

## Methods

### Study Design

The author's previously published model [20], [21] of HIV transmission and disease progression was modified to include acute HIV screening via NAAT. I instantiated the model using demographic, epidemiologic, and cost data for the United States. I then numerically simulated the epidemic over a 20-year time horizon and estimated population-level outcomes, including HIV incidence, AHI identified, quality-adjusted life years (QALYs), costs, and cost-effectiveness. Additional model details are provided as Supporting Information (Text S1).

### Population

To account for variations in behavior and infection risk, the adult population aged 15 to 64 years was subdivided based on gender, risk behavior (MSM, injection drug users (IDU), MSM/IDU, or low-risk), and male circumcision status (Table S1). By integrating data on population sizes, number of people living with HIV, and the distribution of infections by transmission mode, *undiagnosed* HIV prevalence in each risk group was estimated: 4.3% (MSM), 4.4% (male IDUs), 6.4% (MSM/IDUs), 5.9% (female IDUs), 0.03% (low-risk men), and 0.07% (low-risk women).[1], [22]–[28] The HIV-infected population was further divided based on disease stage, identification status, and antiretroviral treatment status. The model included population entry and exit, non-HIV-related mortality, and IDU-related mortality (Table S2).

### HIV Transmission and Progression

An important public health benefit of HIV screening is reduced transmission due to (1) effective counseling aimed at reducing risky behavior, and (2) earlier ART initiation, which suppresses viral load, reducing the chance of transmission.[29]–[35] The model was explicitly designed to capture the population-level benefits of reduced transmission, as well as the individual benefits of reduced disease progression, morbidity, and mortality. The model includes transmission via heterosexual and homosexual contact, and via needle-sharing (Figure S1), and accounts for *secondary transmission* in the population, which is a key advantage of a dynamic model. Proportional mixing was assumed (i.e., individuals with many partners are more likely to select a partner with many partners), and transmission probabilities were varied based on gender, disease stage, and ART status.

I assumed that AHI occurs for two months after initial infection,[6], [36] and that individuals with AHI are ten times as infectious as in the asymptomatic period,[5], [29], [36]–[38] but both assumptions were varied in sensitivity analysis.

### HIV Screening

The model varies the population screened (all adults, MSM and IDUs, or MSM only), frequency (annually or every six months), and test sequence (third- or fourth-generation immunoassay, or immunoassay with pooled NAAT if immunoassay-negative). The latter attribute allows delineation of screening for prevalent infection (via fourth-generation assay), and testing for acute infection (via NAAT). In the “status quo” scenario, current HIV screening rates are assumed to persist for the model's duration (Table 1).

**Table 1:** Key HIV screening parameters.

Parameter	Value	Range	Source
Proportion tested in past 12 months (status quo)			
High-risk individuals	23%	10–30%	[44]
Low-risk individuals	10%	5–20%	[44]
Symptom-based case finding per year			
Symptomatic HIV	10%	0–30%	[35]
AIDS	20%	10–60%	[35]
Window period of detection (days)			
3rd generation ELISA	22	14–40	[6], [7], [10]
4th generation immunoassay	17	10–28	[6], [18]
NAAT	11	9–30	[6], [11]
NAAT pooling algorithm sensitivity	95%	90–100%	[39]
NAAT pooling efficiency (tests/specimen)	0.11	0.10–1.0	[2], [39]
Proportion tested who receive NAAT test results	80%	50–100%	[17]
Reduction in sexual partners if identified	50%	20–70%	[8], [9], [45]
Cost of 3rd-generation ELISA	$15	$10–$25	[11], [35], [43], [46], [47]
Cost of Western Blot confirmatory test	$40	$25–$50	[47]
Cost of NAAT	$120	$100–$150	[11]–[14], [39], [46]
Cost of quantitative viral load assay	$120	$100–$140	[48], [49]
Cost of HIV counseling	$60	$40–$100	[35], [42], [43]

ELISA  =  enzyme linked immunosorbent assay; NAAT  =  nucleic acid amplification test.

#### Immunoassay

The new fourth-generation immunoassay, the Architect HIV Ag/Ab Combo Assay (Abbott Diagnostics, Wiesbaden, Germany), detects antigens on average 17 days (the window period) after infection,[6], [18] although I varied this in sensitivity analysis (Figure S2). Before 17 days, individuals would observe a negative immunoassay test and subsequently be included in a pooled NAAT test, if applicable. After the window period, individuals would receive a positive immunoassay test and receive confirmatory testing via Western Blot; however, they would not receive an AHI diagnosis. Because fourth-generation assays may not be regularly used in all healthcare settings, I also considered expanded screening with a third-generation ELISA.

#### Pooled NAAT

To test for AHI, I assumed use of a qualitative HIV RNA assay, such as the Aptima HIV-1 RNA Assay (Gen-Probe Inc, San Diego, California), and that only persons with a negative immunoassay are tested via pooled NAAT. Post-infection, viremia levels increase exponentially from undetectable to a level detectable by NAAT. I assumed that NAAT positivity occurs 11 days after infection.[6], [11] Given a lower limit of detection of 30–50 copies/mL, the NAAT pooling sensitivity was assumed to be 95% during the period before immunoassay positivity.[39]

To ensure rapid turnaround of test results, I assumed a one-stage pooling algorithm with pools containing ten samples, resulting in an pooling efficiency (i.e., average number of tests per specimen) of 0.11, assuming 0.5% of the HIV-infected population has AHI.[2], [39] Samples from positive pools are then screened individually, and any individual sample with a positive test undergoes a quantitative HIV RNA assay. I assumed that 80% of individuals receive their test results,[17] and that persons diagnosed with AHI receive post-test counseling and linkage to care.

### Health Outcomes and Costs

The mathematical model projects new HIV infections among each risk group, as well as total QALYs for the population. I included the costs of diagnostic services, confirmatory testing, and counseling associated with each screening intervention (Table 1), as well as future HIV-related and non-HIV-related healthcare costs and ART costs, assuming a societal perspective. I assumed that third- and fourth-generation assays cost the same, but I varied this assumption in sensitivity analysis. The incremental cost-effectiveness ratios (ICER) of each screening strategy were calculated, to compare the costs and health benefits of each strategy with the status quo and the next-best strategy on the cost-effectiveness frontier. Importantly, the dynamic HIV epidemic model captures the benefits of reduced secondary transmission in cost-effectiveness estimates. Results are presented as cumulative outcomes over a 20-year time horizon. Costs and QALYs were discounted at an annual rate of 3%, with costs given in 2009 U.S. dollars.[40]

## Results

Annual HIV incidence (and incidence rates) among each population were projected: 28,000 (0.7%) among MSM, 11,700 (1.2%) among IDUs, 5,100 (1.7%) among MSM/IDUs, and 15,500 (0.01%) among low-risk men and women, which are broadly consistent with recent estimates.[23], [25] Under the status quo, an estimated 1.18 million new infections occur over 20 years with approximately 158,000 infections (13.4%) caused by persons with AHI.[3], [5], [41] Variations in the contribution of AHI were examined in sensitivity analysis.

### HIV Infections Prevented

#### Fourth-Generation Immunoassay

Annual screening of all adults with fourth-generation immunoassay prevents 171,562 infections over 20 years (14.5% of the projected total); screening every six months prevents 17.2% (Table 2). Strategies that screen only key populations (MSM and IDUs) prevent a substantial number of new infections: 12.8% if screened annually, or 15.3% if screened every six months. Exclusively screening MSM reduces incidence by 9.5% or 11.4% with annual or semi-annual frequency, respectively. Although the feasibility of a frequent, universal HIV screening program is questionable, these strategies were considered to examine their theoretical benefit on the epidemic.

**Table 2:** Health and economic outcomes over 20 years with 4th-generation immunoassay.

Screening Strategy	AHI identified with NAAT per year	HIV infections prevented over 20 years (% of status quo)	Incremental costs (billions)	Incremental QALYs (millions)	ICER ($/QALY)	
					vs status quo	vs next best
Screen all adults						
4G assay only						
Annually	---	−171,562 (14.5%)	$229.3	2.32	$98,700	$98,700
Every 6 months	---	−203,677 (17.2%)	$471.3	2.74	$172,200	$583,000
4G assay & NAAT						
Annually	817	−172,791 (14.6%)	$271.6	2.34	$116,300	$3,174,000*
Every 6 months	1,427	−205,360 (17.4%)	$556.0	2.76	$201,800	$4,700,600
Screen MSM and IDUs						
4G assay only						
Annually	---	−150,778 (12.8%)	$11.9	1.87	$6,400	$6,400
Every 6 months	---	−181,278 (15.3%)	$18.7	2.25	$8,300	$17,800
4G assay & NAAT						
Annually	623	−151,949 (12.9%)	$12.9	1.88	$6,900	$80,300*
Every 6 months	1,094	−182,887 (15.5%)	$20.7	2.27	$9,100	$117,436
Screen MSM						
4G assay only						
Annually	---	−113,142 (9.5%)	$8.0	1.37	$5,800	$5,800
Every 6 months	---	−135,147 (11.4%)	$12.9	1.64	$7,900	$18,300
4G assay & NAAT						
Annually	410	−113,939 (9.6%)	$8.8	1.38	$6,400	$92,200*
Every 6 months	708	−136,223 (11.5%)	$14.5	1.65	$8,800	$138,200

All outcomes are relative to the status quo (approximately 1.18 million new infections over 20 years). MSM  =  men who have sex with men; IDU  =  injection drug user; 4G assay  =  fourth-generation antigen-antibody combination assay; AHI  =  acute HIV infection; NAAT  =  nucleic acid amplification test; QALY  =  quality-adjusted life year; ICER  =  incremental cost-effectiveness ratio compared to the status quo or next best strategy for each risk group. *  =  weakly dominated strategy (this strategy is not on the cost-effectiveness frontier and is thus not economically efficient).

#### Immunoassay and NAAT

Augmenting universal screening with pooled NAAT prevents few additional infections over 20 years: 1,229 (0.1%) if testing annually or 1,683 (0.2%) if testing every six months. Targeted NAAT testing of MSM and IDUs prevents 1,171 to 1,609 infections, and screening only MSM prevents 797 to 1,076 infections over 20 years. Because more than 50% of new infections in the United States occur among MSM, targeted screening MSM prevents nearly two-thirds as many HIV infections as universal testing. Of note, annual NAAT testing prevents substantially fewer infections than a more frequent semi-annual screening program with fourth-generation immunoassay only.

#### Third-Generation ELISA

In general, expanded screening with third-generation ELISA prevents slightly fewer new HIV cases than with fourth-generation immunoassay (Table 3). For example, annual screening of all adults with fourth-generation assay prevents 171,562 infections over 20 years, compared to 171,071 with third-generation ELISA, a difference of less than 0.3%.

**Table 3:** Health and economic outcomes over 20 years with 3rd-generation ELISA.

Screening Strategy	AHI identified with NAAT per year	HIV infections prevented over 20 years (% of status quo)	Incremental costs (billions)	Incremental QALYs (millions)	ICER ($/QALY)	
					vs status quo	vs next best
Screen all adults						
3G ELISA only						
Annually	---	−171,071 (14.5%)	$229.3	2.32	$99,000	$99,000
Every 6 months	---	−203,295 (17.2%)	$471.3	2.74	$172,400	$581,000
3G ELISA & NAAT						
Annually	1,501	−173,340 (14.7%)	$271.6	2.34	$116,000	$1,719,000*
Every 6 months	2,627	−206,409 (17.5%)	$556.0	2.77	$201,000	$2,540,300
Screen MSM and IDUs						
3G ELISA only						
Annually	---	−150,286 (12.7%)	$11.9	1.86	$6,400	$6,400
Every 6 months	---	−180,889 (15.3%)	$18.7	2.25	$8,300	$17,700
3G ELISA & NAAT						
Annually	1,145	−152,449 (12.9%)	$12.9	1.89	$6,900	$43,500*
Every 6 months	2,014	−183,867 (15.6%)	$20.7	2.28	$9,100	$63,179
Screen MSM						
3G ELISA only						
Annually	---	−112,649 (9.5%)	$8.0	1.37	$5,800	$5,800
Every 6 months	---	−134,730 (11.4%)	$12.9	1.64	$7,900	$18,200
3G ELISA & NAAT						
Annually	754	−114,121 (9.7%)	$8.8	1.38	$6,300	$49,600*
Every 6 months	1,303	−136,719 (11.6%)	$14.5	1.66	$8,700	$74,100

All outcomes are relative to the status quo (approximately 1.18 million new infections over 20 years). MSM  =  men who have sex with men; IDU  =  injection drug user; 3G ELISA  =  third-generation enzyme linked immunosorbent assay; AHI  =  acute HIV infection; NAAT  =  nucleic acid amplification test; QALY  =  quality-adjusted life year; ICER  =  incremental cost-effectiveness ratio compared to the status quo or next best strategy for each risk group. *  =  weakly dominated strategy (this strategy is not on the cost-effectiveness frontier and is thus not economically efficient).

### Acute Infections Identified

#### NAAT

As newer immunoassays become widely used, the number of AHI identified via NAAT diminishes because the newer assay (fourth-generation) identifies more infected persons than the older test (third-generation ELISA) (Tables 2 and 3). For example, annual NAAT testing of MSM and IDUs identifies 623 AHI per year, assuming NAAT follows a fourth-generation assay. If third-generation ELISA is instead utilized, then NAAT detects 1,145 AHI. Screening twice as often (every six months) identifies approximately 75% more AHI. This strategy has decreasing returns because annual testing reduces overall incidence; hence, there are fewer persons with AHI to identify six months later. A similar effect occurs with screening every three months.

#### Immunoassay Only

In settings where NAAT is not utilized, screening with only a fourth-generation assay identifies more persons as HIV+ than with third-generation ELISA, because the newer test detects antigen/antibodies earlier. For example, annual fourth-generation testing of MSM and IDUs detects approximately 400 additional HIV+ persons per year, compared to screening with third-generation ELISA.

### Cost-Effectiveness Analysis

Universal HIV screening with a fourth-generation immunoassay adds 2.3 to 2.7 million QALYs over 20 years at a cost of $100,000 to $580,000 per QALY gained (Table 2). Targeted screening of MSM and IDUs or MSM only offers more favorable cost-effectiveness ratios: annual testing with immunoassay costs less than $10,000 per QALY gained; screening every six months costs less than $20,000 per QALY gained.

Because detection of AHI among low-risk populations is uncommon, universal NAAT testing of all adults costs $3.2 million to $4.7 million per QALY gained, which far exceeds any acceptable cost-effectiveness threshold. Annual NAAT screening of MSM and IDUs or only MSM costs $80,000 to $92,000 per QALY gained, which is generally considered cost-effective. Semi-annual NAAT testing is less economically efficient, at a cost of up to $140,000 per QALY gained. Of note, the analysis suggests that screening MSM with fourth-generation immunoassay every six months is more cost-effective than less frequent, annual testing with NAAT.

**Figure 1:**
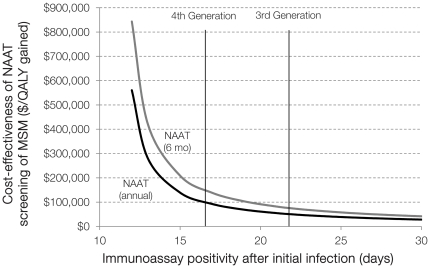
One-way sensitivity analysis on the window period of detection. The cost-effectiveness of NAAT testing of MSM, given variations in the window period of detection for the immunoassay. Testing occurs annually (black line) or every six months (grey line), and cost-effectiveness is relative to screening with immunoassay only. MSM  =  men who have sex with men; NAAT  =  nucleic acid amplification test; QALY  =  quality-adjusted life year.

**Figure 2:**
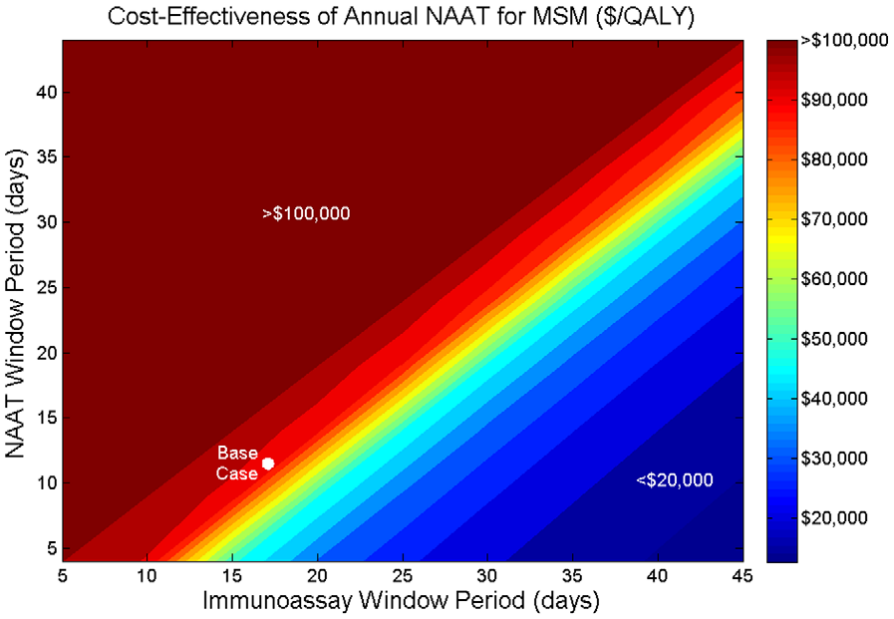
Two-way sensitivity analysis on the window period of detection. The cost-effectiveness of annual NAAT testing of MSM, given simultaneous variations in the window periods of detection for the immunoassay and NAAT, assuming the immunoassay detects infection after NAAT. Cost-effectiveness is relative to annual screening with immunoassay only. MSM  =  men who have sex with men; NAAT  =  nucleic acid amplification test; QALY  =  quality-adjusted life year.

Compared to screening with third-generation ELISA, pooled NAAT is more cost-effective because of the longer window period between NAAT and ELISA positivity (Table 3, Figures 1 and 2). Annual NAAT testing of MSM costs $50,000 per QALY gained in settings where third-generation ELISA is the dominant alternative, but $92,000 per QALY gained relative to fourth-generation testing.

### Sensitivity Analysis

**Figure 3:**
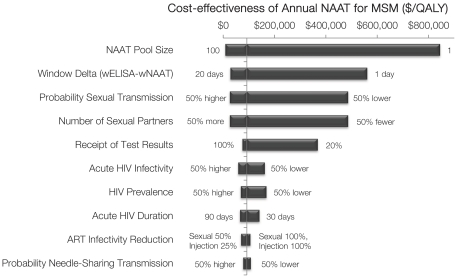
Tornado diagram with variations in model parameters. One-way sensitivity analysis of model parameters, where each horizontal bar shows the range in cost-effectiveness given variations in each parameter value. The vertical line shows the base case cost-effectiveness ($92,000 per QALY gained). Cost-effectiveness of annual NAAT testing of MSM is relative to annual screening with fourth-generation immunoassay only. MSM  =  men who have sex with men; NAAT  =  nucleic acid amplification test; QALY  =  quality-adjusted life year; ART  =  antiretroviral therapy; window delta  =  days between NAAT and immunoassay positivity.

I evaluated variations in all model parameters, and the following parameters most significantly affected results (Figure 3).

#### Window Period

I initially assumed that fourth-generation assays detect infection after 17 days, but this window period is uncertain. As newer tests shorten the time between initial infection and test positivity, the relative benefits of NAAT decrease, which worsens cost-effectiveness estimates (Figure 1). The cost-effectiveness of NAAT improves as the immunoassay window period increases, or as the NAAT window period decreases (Figure 2).

### 4.2 MSM HIV Prevalence

**Figure 4:**
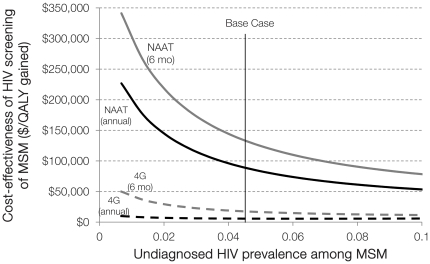
One-way sensitivity analysis on undiagnosed HIV prevalence among MSM. The cost-effectiveness of NAAT (solid line) or fourth-generation immunoassay (dashed line) testing of MSM, given variations in undiagnosed HIV prevalence among MSM. Testing occurs annually (black line) or every six months (grey line), and cost-effectiveness of NAAT is relative to screening with fourth-generation immunoassay only. MSM  =  men who have sex with men; NAAT  =  nucleic acid amplification test; 4G  =  fourth-generation immunoassay; QALY  =  quality-adjusted life year.

Undiagnosed HIV prevalence among MSM was estimated to be 4.3% (with 12.5% overall HIV prevalence). As prevalence decreases, the cost-effectiveness of NAAT worsens (Figure 4). If undiagnosed HIV prevalence is only 1%, annual NAAT screening identifies 160 AHI in MSM per year, at a cost of $200,000 per QALY gained. In settings where undiagnosed HIV prevalence exceeds 10%, annual NAAT testing of MSM costs less than $50,000 per QALY gained, and semi-annual screening costs less than $80,000 per QALY gained.

#### Impact of Acute HIV Infection

The base case conservatively estimated that AHI accounts for 13% of new HIV infections in the U.S. If the relative contribution of AHI is greater, due to a longer duration of AHI or greater infectivity during this period, the cost-effectiveness of NAAT improves, and vice versa (Table 4, Figure 3). For example, a three-month duration results in 20% of new cases due to AHI, and annual NAAT screening of MSM costing $64,300 per QALY gained (base case: $92,200).

**Table 4:** Sensitivity analysis on the impact of acute HIV infection.

	Duration of AHI	Fraction of new HIV infections due to AHI	HIV infections prevented over 20 years (% of status quo)		ICER ($/QALY)	
Scenario			Screen MSM annually	Screen MSM every 6 months	Screen MSM annually	Screen MSM every 6 months
Base Case	2 months	13.4%	9.6%	11.5%	$92,200	$138,200
Longer duration of AHI	3 months	20.0%	9.9%	11.8%	$64,300	$95,400
Shorter duration of AHI	1 month	6.7%	9.3%	11.1%	$141,200	$224,000
50% greater infectivity during AHI	2 months	17.9%	10.3%	12.3%	$57,500	$81,300
50% lower infectivity during AHI	2 months	8.7%	9.0%	10.7%	$162,000	$276,400
Longer duration of AHI & 50% greater infectivity	3 months	26.4%	11.1%	13.3%	$34,000	$48,500
Shorter duration of AHI & 50% lower infectivity	1 month	4.3%	9.0%	10.8%	$198,100	$358,300

All scenarios assume MSM are screened with a fourth-generation immunoassay and pooled NAAT.

MSM  =  men who have sex with men; AHI  =  acute HIV infection; NAAT  =  nucleic acid amplification test; QALY  =  quality-adjusted life year; ICER  =  incremental cost-effectiveness ratio compared to screening with fourth-generation immunoassay only.

#### Receipt of Test Results

**Figure 5:**
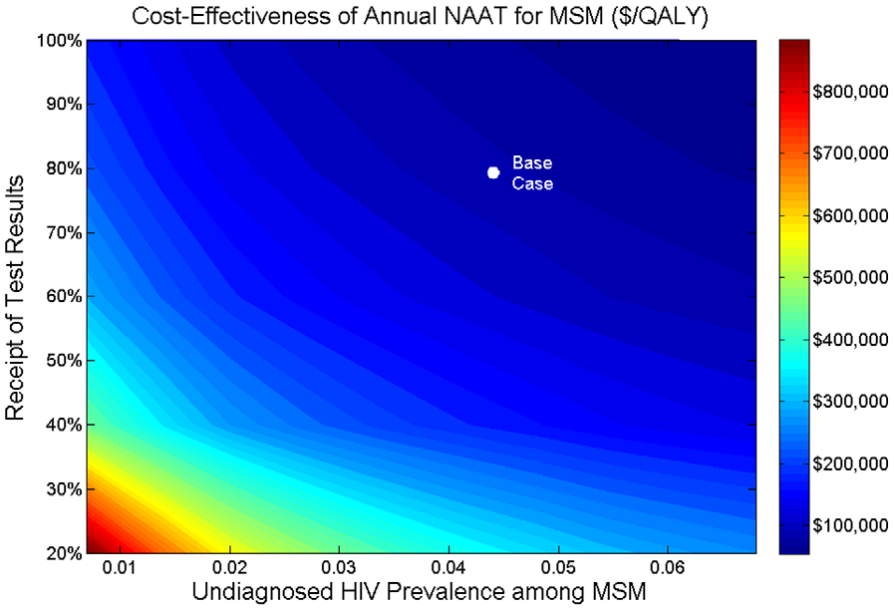
Two-way sensitivity analysis on undiagnosed HIV prevalence among MSM. The cost-effectiveness of annual NAAT testing of MSM, given simultaneous variations in undiagnosed HIV prevalence among MSM and the proportion receiving NAAT test results. Cost-effectiveness is relative to screening with fourth-generation immunoassay only. MSM  =  men who have sex with men; NAAT  =  nucleic acid amplification test; QALY  =  quality-adjusted life year.

The base case assumed that 80% of persons tested for AHI receive their test results. If only 50% of MSM receive their results with annual NAAT testing, only 250 AHI are identified per year, for $147,000 per QALY gained (Figure 5). With only a 20% test receipt, this strategy exceeds $350,000 per QALY gained and is not economically efficient.

#### Behavior Change

A minimal reduction in risky sexual behavior following testing and counseling attenuates some of the benefits of expanded screening. With a 20% reduction in behavior, the annual NAAT screening of MSM exceeds $130,000 per QALY gained; with no reduction in behavior, the strategy exceeds $600,000 per QALY gained.

#### Price of Fourth-Generation Immunoassay

Given a price of $50 or $100, annual immunoassay testing of MSM costs $16,000 or $30,000 per QALY gained, respectively. At approximately $315 per test, screening with fourth-generation tests or pooled NAAT have similar cost-effectiveness.

#### Early ART Initiation

The recent HPTN 052 trial indicated that early ART initiation can substantially reduce HIV transmission in sero-discordant couples, and prior studies suggest that augmenting a routine HIV screening program with increased treatment improves health outcomes.[20] Although early ART initiation is beyond the focus of this study, I estimated that an annual screening program (via fourth-generation assay) for all adults, coupled with 75% treatment initiation of all HIV+ persons could avert 50-60% of projected future HIV cases. Adding pooled NAAT prevents an additional 0.1% of cases, a very modest improvement, because most of the benefit accrues from maintaining high treatment coverage levels. This suggests that a more sophisticated analysis of early treatment access, attrition, adherence, and possible resistance should be performed.

## Discussion

The present study estimated the population-level health benefits and costs of alternative acute HIV screening strategies in the U.S., and the results highlight several important findings. This study is the first to estimate the cost-effectiveness of expanded AHI screening to all adults or key populations only, and to consider expanded screening with fourth-generation immunoassay versus pooled NAAT testing.

First, screening MSM annually with a fourth-generation assay prevents 5,700 infections per year (9.5% of the total) for less than $10,000 per QALY gained. Adding a pooled NAAT test following a negative-immunoassay identifies 410 acute infections per year, but reduces HIV incidence only modestly, for more than $92,000 per QALY gained. Doubling the NAAT testing frequency to every six months identifies 75% more acute HIV infections but with a cost-effectiveness exceeding $138,000 per QALY gained. Alternatively, semi-annual screening with immunoassay only leads to an overall greater increase in health benefits and is more economically efficient than annual NAAT testing. Screening MSM every six months reduces annual HIV incidence by 6,800, adds 1.6 million QALYs to the population over 20 years, for less than $20,000 per QALY gained. These results underscore that semi-annual HIV screening via fourth-generation immunoassay is a better use of resources than annual pooled NAAT.

Second, because MSM account for only 4% of the male population but 68% of HIV cases among men,[24] targeted HIV screening every six or 12 months is a particularly cost-effective strategy. Screening IDUs as well prevents more infections with similar cost-effectiveness ratios, although the uptake of frequent screening among IDUs may be less feasible. Screening all adults once per year reduces annual incidence by 15%, but this strategy is less cost-effective than targeted screening, which is consistent with prior studies.[35], [42], [43] The results suggest that even the most optimistic HIV screening program where everyone is screened at least once per year, prevents less than 20% of new infections and is not sufficient to eliminate the HIV epidemic in the United States. Although likely infeasible, I considered such a hypothetical strategy to compare the efficiency of annual or semi-annual HIV testing of all adults versus IDUs and/or MSM only. Augmenting routine HIV screening with increased antiretroviral treatment improves epidemic outcomes,[20] and earlier initiation of treatment further reduces new cases.

Third, the cost-effectiveness of acute HIV screening depends on each test's window period of detection after initial infection. As fourth-generation immunoassays become widely available, routine testing of MSM with pooled NAAT is less cost-effective, assuming the price of an immunoassay does not increase. If third-generation ELISA tests remain the predominant alternative, pooled NAAT may offer a valuable public health benefit, costing less than $75,000 per QALY gained with semi-annual screening.

A key factor driving the favorable cost-effectiveness ratios associated with screening of MSM is high HIV prevalence among this population, and by extension, high HIV incidence. At undiagnosed HIV prevalence levels below 4%, annual NAAT testing of MSM exceeds $100,000 per QALY gained. Policymakers aiming to allocate limited resources most effectively should consider the local epidemic's characteristics. If HIV prevention programs, such as education campaigns, condom distribution, and partnership notification, are shown to be effective in very low prevalence settings, it may be optimal to continue investing in such programs rather than scale-up testing.

The model projected that 13% of new cases are attributable to acute HIV infection, although this contribution is uncertain,[4] and cost-effectiveness improves dramatically as the relative impact of AHI increases. If one-quarter of new cases is attributable to AHI, then semi-annual NAAT testing of MSM costs less than $50,000 per QALY gained. If AHI contributes to only 5% of HIV cases, then NAAT testing costs $200,000 to $350,000 per QALY gained.

Finally, the results suggest that efforts to reduce risky sexual behavior following identification of AHI should be emphasized, as failure to do so worsens cost-effectiveness estimates. To achieve the full potential benefits of AHI screening, public health departments, practitioners, and community-based clinics offering NAAT testing should include concomitant efforts to counsel individuals with risk behavior reduction and partner notification.

This study complements other recent HIV screening cost-effectiveness studies. Hutchinson et al. found that the cost-effectiveness of pooled NAAT versus third-generation ELISA depends substantially on local HIV prevalence levels.[17] Although the present analysis includes the entire U.S. population and fourth-generation immunoassay, the results are broadly consistent. Two independent prior studies evaluated the cost-effectiveness of HIV screening in the U.S., although the baseline incidence, undiagnosed HIV prevalence, and test characteristics differed from the current study.[35], [43] Sanders et al. found that a routine screening program prevented 21% of secondary cases,[35] and Paltiel et al. estimated that annual screening reduced incidence by 5,100 out of 44,000–60,000,[43] which are in line with the present study's estimate of 10–15% depending on the target population. Additionally, both studies only approximated reduced secondary HIV transmission, which my dynamic transmission model is explicitly designed to estimate.

The current modeling study has several key limitations. As with most epidemic models, I simplified the complex dynamics of disease transmission and partnership selection. Although I stratified the population based on risk-status, I did not adopt a more granular categorization, to maintain computational feasibility. I evaluated early ART initiation only in sensitivity analysis, and given the high viremia levels during this period, ART initiation during AHI may be a cost-effective strategy and further investigation is warranted. I did not explicitly model partner notification following AHI identification, although if individuals do refer partners to be tested, this would likely improve cost-effectiveness estimates. Finally, I ignored the potential effects of sero-sorting after identification, which would again make the results appear more favorable.

Quantifying the potential health benefits of pooled NAAT testing for acute HIV infection is an important question as use of newer antigen-antibody immunoassays becomes increasingly widespread. In settings where undiagnosed HIV prevalence levels exceed 4% among MSM, annual NAAT testing for acute HIV infection costs less than $100,000 per QALY gained. However, screening every six months with a fourth-generation immunoassay is a more economically efficient use of program resources. Augmenting acute HIV testing with early initiation of antiretroviral treatment may further justify the use of pooled NAAT testing, and the cost-effectiveness of such a strategy should be explored.

## Supporting Information

Text S1 **Technical appendix with additional model details and parameterization.**
(PDF)

Figure S1 **Schematic diagram of HIV transmission model and potential modes of transmission.** The boxes represent cohorts of individuals in each disease stage and the arrows represent transitions due to disease transmission, disease progression, mortality, screening, or treatment initiation. ART  =  antiretroviral therapy. A description of each parameter is given in Table S2.
(JPG)

Figure S2 **Window period of detection during acute HIV.** The figure shows the duration (in days) of the acute infection period (*1/θ_ACUTE_*), and the window period of detection for each test: nucleic acid amplification test (*ω_NAAT_*), fourth-generation immunoassay (*ω_4GEN_*), and third-generation enzyme linked immunosorbent assay (*ω_3GEN_*).
(JPG)

Table S1 **Modes of HIV transmission.**
(PDF)

Table S2 **Summary of key model parameters.**
(PDF)
