# Supplementary material for: HIV Screening via Fourth-Generation Immunoassay or Nucleic Acid Amplification Test in the United States: A Cost-Effectiveness Analysis
Source: PLoS One. 2011 Nov 16;6(11):e27625. doi: 10.1371/journal.pone.0027625 (PMC3218000; doi:10.1371/journal.pone.0027625)
Supplement: Text S1 — Technical appendix with additional model details and parameterization. (PDF) [file pone.0027625.s001.pdf]

HIV Screening via Fourth-Generation Antigen-Antibody or Nucleic Acid Amplification Test: A Cost-Effectiveness Analysis

Supporting Information

Elisa F. Long, Ph.D.

Yale University

elisa.long@yale.edu

This dynamic compartmental model captures HIV disease progression and HIV transmission via heterosexual contact, homosexual contact, and needle-sharing, under varying levels of HIV screening and treatment with antiretroviral therapy. The current model is an extension of the author's previously published HIV transmission model.[1, 2]

S.1 HIV Epidemic Model

S.1.1 Risk Groups

The adult population was subdivided into six risk groups (male IDU, male MSM, male IDU/MSM, male other, female IDU, female other). This particular set of risk groups was selected to capture variations in demographics (population sizes, initial HIV prevalence, mortality rate), behavior (number of sexual partners, condom use, injection drug use, needle-sharing), as well as known epidemiological factors (probability of disease transmission, effect of male circumcision).

S.1.2 Transmission Modes

The dynamic model captures HIV transmission via three modes: heterosexual contact, homosexual contact, and needle-sharing. Table S1 shows the possible modes of transmission between any two risk groups. In the model, men who have sex with men were allowed to have heterosexual contact with women.

S.1.3 Disease Transmission

The sufficient contact rate between uninfected and infected individuals is represented as a matrix, $\lambda = [\lambda_{i,j}]$, where $\lambda_{i,j}$ represents the sufficient contact rate between members of (uninfected) compartment i and members of (infected) compartment j. I calculated the total contact rate, $\lambda_{i,j}$, as the sum of the three transmission modes: needle-sharing ($\gamma_{i,j}$), opposite-sex (heterosexual) contact ($\beta^o_{i,j}$), and same-sex (homosexual) contact ($\beta^s_{i,j}$).

The sufficient contact rates due to needle-sharing, opposite-sex, and same-sex contact were modeled as binomial processes, where a "success" is defined as infection transmission. Uninfected individuals randomly select a partner n times; the probability of "success" is the probability of transmission per partnership.

$$
\begin{aligned}
P\{\mathrm{T}\} &= 1 - P\{\text{no T}\} \\
&= 1 - \left(P\{\text{no T per trial}\}\right)^{\#\text{ trials}} \\
&= 1 - \left(1 - P\{\text{T per trial}\}\right)^{\#\text{ trials}} \\
&= 1 - \left(1 - P\{\text{select person in } j\}\, P\{\text{T per trial} \mid \text{select person in } j\}\right)^{\#\text{ trials}}
\end{aligned}
$$

where T refers to disease transmission and a trial is either a sexual partnership or shared needle.

Needle-sharing transmission The needle-sharing sufficient contact rate between uninfected individuals in compartment i and infected individuals in compartment j is:

$$\gamma_{i,j}(t) = 1 - \left(1 - \left[\frac{X_j(t) d_j s_j}{\sum_k X_k(t) d_k s_k}\right] \tau_{i,j}\right)^{d_i s_i} \tag{S.1}$$

where i, j, k correspond to compartments of IDUs. The term in brackets, $\left[\frac{X_j(t) d_j s_j}{\sum_k X_k(t) d_k s_k}\right]$, corresponds to the probability of selecting a needle-sharing partner in compartment j, based on a proportional mixing assumption (i.e., individuals with many partners are more likely to select a partner who also has many partners). The probability of needle-sharing transmission, $\tau_{i,j}$, between individuals in compartment i and j depends on the transmission probability per shared needle, π^k, and the reduction in infectivity due to ART, δ_h^d (if individuals in compartment j are receiving ART).

Heterosexual transmission The opposite-sex (heterosexual) sufficient contact rate between uninfected individuals in compartment i and infected individuals in compartment j

is:

$$\beta^o_{i,j}(t) = 1 - \left(1 - \left[\frac{X_j(t) n^o_j (1-u^o_j \kappa)}{\sum_k X_k(t) n^o_k (1-u^o_k \kappa)}\right] \sigma_{i,j}\right)^{n^o_i (1-u^o_i \kappa)} \tag{S.2}$$

where i is male and j, k are female, or i is female and j, k are male. The term in brackets, $\left[\frac{X_j(t) n^o_j (1-u^o_j \kappa)}{\sum_k X_k(t) n^o_k (1-u^o_k \kappa)}\right]$, corresponds to the probability of selecting a sexual partner in compartment j. The probability of heterosexual transmission, $\sigma_{i,j}$, between individuals in compartment i and j depends on the transmission probability per partnership, π^k_{mf} or π^k_{fm} (which reflects each partners' gender), and the reduction in infectivity due to ART, δ^s_h. For men, $\sigma_{i,j}$ was also adjusted by $1 - \delta_c$ based on circumcision status. Additionally, the number of sexual partners for status-aware infected individuals in compartment j was adjusted by $1 - \epsilon_k$.

Homosexual transmission The same-sex (homosexual) sufficient contact rate between uninfected individuals in compartment i and infected individuals in compartment j is:

$$\beta^s_{i,j}(t) = 1 - \left(1 - \left[\frac{X_j(t) n^s_j (1-u^s_j \kappa)}{\sum_k X_k(t) n^s_k (1-u^s_k \kappa)}\right] \sigma_{i,j}\right)^{n^s_i (1-u^s_i \kappa)} \tag{S.3}$$

where i, j, k correspond to compartments of MSM. The term in brackets, $\left[\frac{X_j(t) n^s_j (1-u^s_j \kappa)}{\sum_k X_k(t) n^s_k (1-u^s_k \kappa)}\right]$, again corresponds to the probability of selecting a sexual partner in compartment j. As with heterosexual transmission, the probability of homosexual transmission, $\sigma_{i,j}$, depends on the transmission probability per partnership, π^k_{mm}, and the reduction in infectivity due to ART, δ^s_h. In the model, male circumcision was assumed to have no effect on homosexual transmission, although this assumption can be updated as additional clinical data become available. Once again, status-aware infected individuals reduce their number of sexual partners by ϵ_k.

Total transmission I calculated the overall sufficient contact rate between uninfected individuals in compartment i and infected individuals in compartment j by first converting the annual transmission probability to a continuous rate, according to the formula $rate =$

$-ln(1-p)/t$. The total contact rate was calculated as the sum of the three modes of transmission: needle-sharing ($\gamma_{i,j}$), opposite-sex (heterosexual) contact ($\beta^o_{i,j}$), and same-sex (homosexual) contact ($\beta^s_{i,j}$). For small probability values, the approximation $p \approx -ln(1-p)$ was assumed. The total contact rate at time t between individuals in compartments i and j, $\lambda_{i,j}(t)$, is:

$$\begin{aligned}\lambda_{i,j}(t) &= -ln[1-\gamma_{i,j}(t)] + -ln[1-\beta^o_{i,j}(t)] + -ln[1-\beta^s_{i,j}(t)] \\ \lambda_{i,j}(t) &\approx \gamma_{i,j}(t) + \beta^o_{i,j}(t) + \beta^s_{i,j}(t) \qquad \text{(S.4)}\end{aligned}$$

S.2 HIV Interventions

I compared alternative HIV screening strategies by varying the following attributes:

- Targeted risk group (everyone, MSM and IDUs, or MSM only)
- Screening frequency (annually, every six months, every three months)
- Tests offered (immunoassay only, or immunoassay followed by pooled NAAT if immunoassay-negative)

S.2.1 HIV Screening

It was assumed that voluntary HIV screening was accompanied by an effective counseling program may help reduce an individual's number of heterosexual partners (n^o_j) and homosexual partners (n^s_j), which subsequently reduces the sufficient contact rate (Equations S.2-S.3). The degree of behavior change (ε_k) was allowed to vary by HIV status, where k refers to acute HIV or chronic infection (asymptomatic, symptomatic, or AIDS).

Unidentified (i.e., status-unaware) individuals with *acute HIV infection* in compartment i transition to compartment $i+1$ at rate ψ_i, which depends on the rate of HIV screening via immunoassay (ψ_{ASSAY}) or NAAT (ψ_{NAAT}), as well as the test's sensitivity at detecting

infection:

$$\psi_i = f_{NAAT} \cdot sens_{NAAT} \left(\frac{\omega_{ASSAY}}{1/\theta_{ACUTE}} - \frac{\omega_{NAAT}}{1/\theta_{ACUTE}} \right) \psi_{ACUTE} + \left(1 - \frac{\omega_{ASSAY}}{1/\theta_{ACUTE}} \right) \psi_{ASSAY} \tag{S.5}$$

The terms ω_{ASSAY} (either ω_{3GEN} or ω_{4GEN}) and ω_{NAAT} are the window periods of detection for third- or fourth-generation immunoassay and NAAT, respectively, where $\omega_{ASSAY} > \omega_{NAAT}$ (Figure S1) The average duration of the acute infection period is $1/\theta_{acute}$. The term $\left(\frac{\omega_{ASSAY}}{1/\theta_{ACUTE}} - \frac{\omega_{NAAT}}{1/\theta_{ACUTE}} \right)$ refers to the fraction of individuals with acute infection who would receive a positive NAAT test but a negative immunoassay. The pooling algorithm sensitivity ($sens_{NAAT}$) for the NAAT test depends on the prevalence of acute infection and master pool size. The fraction of individuals who receive their NAAT test results (f_{NAAT}) reduces the overall flow of individuals to the identified compartment. Because all individuals were assumed to receive an immunoassay prior to a NAAT test, the term $\left(1 - \frac{\omega_{ASSAY}}{1/\theta_{ACUTE}} \right)$ refers to the fraction of individuals with acute infection who would receive a positive immunoassay test, and hence would not subsequently receive a NAAT test. The model also assumed that $\psi_{ASSAY} \geq \psi_{NAAT}$, which implies that individuals will always receive an immunoassay test prior to a NAAT test; however, I also consider an "fourth-generation immunoassay only" strategy, where screening via immunoassay was scaled up, but $\psi_{NAAT} = 0$.

Similarly, unidentified individuals with *chronic HIV infection* transition to an identified compartment at rate ψ_{ASSAY}. Finally, individuals with chronic infection may become identified through symptom-based case finding, at rate ν_i, which varies based on disease state (asymptomatic HIV, symptomatic HIV, or AIDS).

S.2.2 HIV Treatment

In the present study, individuals with symptomatic HIV or AIDS are eligible to begin ART regimens. A fraction (ϕ_i) begin ART immediately after identification (via screening or symptom-based case finding), or upon becoming eligible (i.e., advancing from asymptomatic

to symptomatic HIV). Additionally, individuals initiate ART at a continuous rate (α_i) after becoming eligible for treatment.

To model the effects of antiretroviral therapy on health and economic outcomes, I adjusted the appropriate model parameters to account for changes in disease progression rates (θ_i), mortality rates (μ_i), and quality-of-life factors (q_i). I assumed that suppressive antiretroviral therapy reduces an individual's viral load, which reduces the probability of HIV transmission via sexual contact ($\sigma_{i,j}$) and needle-sharing ($\tau_{i,j}$). The model accounted for the direct cost of antiretroviral therapy (c_H), as well as the indirect costs through reduced HIV-related healthcare costs (c_i).

S.3 Dynamic Compartmental Model

To estimate the projected HIV epidemic over time under various HIV screening and treatment scenarios, I created the following system of nonlinear differential equations for each of the six risk groups. Additionally, all male risk groups (male IDU, male MSM, male IDU/MSM, male other) are further subdivided to indicate circumcision status. The complete model comprises 120 equations (4 male groups $\times$ 24 compartments + 2 female groups $\times$ 12 compartments). For compactness, the equations for only one risk group are shown. The remaining five risk groups utilize similar equations, with modified indices. Note, HIV transmission can occur both within and across risk groups according to the appropriate rates of transmission (Equations S.1-S.3).

$$\frac{dX_1}{dt} = \rho_1 \sum_{\forall i} X_i - \psi_1 X_1 - \left(\sum_{j \geq 3} \lambda_{1,j}(t) \right) X_1 - \mu_1 X_1 \quad \text{(S.6)}$$

$$\frac{dX_2}{dt} = \psi_1 X_1 - \left(\sum_{j \geq 3} \lambda_{2,j}(t) \right) X_2 - \mu_2 X_2 \quad \text{(S.7)}$$

$$\frac{dX_3}{dt} = \left(\sum_{j \geq 3} \lambda_{1,j}(t) \right) X_1 + \left(\sum_{j \geq 3} \lambda_{2,j}(t) \right) X_2 - \psi_3 X_3 - \theta_3 X_3 - \mu_3 X_3 \quad \text{(S.8)}$$

$$\frac{dX_4}{dt} = \psi_3 X_3 - \theta_4 X_4 - \mu_4 X_4 \quad \text{(S.9)}$$

$$\frac{dX_5}{dt} = \theta_3 X_3 - \psi_5 X_5 - \theta_5 X_5 - \mu_5 X_5 \quad \text{(S.10)}$$

$$\frac{dX_6}{dt} = \theta_4 X_4 + \psi_5 X_5 - \theta_6 X_6 - \mu_6 X_6 \quad \text{(S.11)}$$

$$\frac{dX_7}{dt} = \theta_5 X_5 - (\psi_7 + \nu_7) X_5 - \theta_7 X_7 - \mu_7 X_7 \quad \text{(S.12)}$$

$$\frac{dX_8}{dt} = \theta_6 (1 - \phi_6) X_6 + (\psi_7 + \nu_7)(1 - \phi_7) X_7 - \alpha_8 X_8 - \mu_8 X_8 \quad \text{(S.13)}$$

$$\frac{dX_9}{dt} = \theta_6 \phi_6 X_6 + (\psi_7 + \nu_7) \phi_7 X_7 + \alpha_8 X_8 - \mu_9 X_9 \quad \text{(S.14)}$$

$$\frac{dX_{10}}{dt} = \theta_7 X_7 - (\psi_{10} + \nu_{10}) X_8 - \mu_{10} X_{10} \quad \text{(S.15)}$$

$$\frac{dX_{11}}{dt} = \theta_8 X_8 + (\psi_{10} + \nu_{10})(1 - \phi_{10}) X_{10} - \alpha_{11} X_{11} - \mu_{11} X_{11} \quad \text{(S.16)}$$

$$\frac{dX_{12}}{dt} = \theta_9 X_9 + (\psi_{10} + \nu_{10}) \phi_{10} X_{10} + \alpha_{11} X_{11} - \mu_{12} X_{12} \quad \text{(S.17)}$$

For ease of notation, let X_i denote $X_i(t)$. A summary of all model parameters is given in Table S2. Figure S2 shows a schematic representation of the model. In the top diagram, boxes represent cohorts of individuals, stratified by HIV status, identification (i.e., screening) status, and treatment status if infected. Arrows represent transitions between compartments. Individuals may also leave each compartment according to the mortality or maturation rate.

S.4 Model Instantiation

The system of nonlinear differential equations (S.6-S.17) was instantiated with initial conditions using 2008 data on population sizes and HIV prevalence levels among each risk group. I divided the HIV-infected population into the four health states (acute HIV, asymptomatic HIV, symptomatic HIV, AIDS) in proportion to the average time spent in each state. The fraction of individuals in each state was then adjusted to account for the increase in life expectancy among individuals with symptomatic HIV and AIDS who are receiving anti-retroviral therapy. I also estimated the fraction of men who are circumcised and assumed this remained constant over the duration of the model's time horizon. The model was implemented in the mathematical programming language Matlab R2010b.

S.5 Model Outcomes

I numerically solved the system of nonlinear differential equations to calculate the number of individuals in each compartment over time. The following outcome measures were calculated: HIV prevalence, new HIV infections, discounted costs and health benefits (quality-adjusted life years experienced), and incremental cost-effectiveness ratios.

HIV prevalence was calculated for each of the six risk groups (male IDU, male MSM, male IDU/MSM, male other, female IDU, female other) as follows:

$$\text{HIV prevalence at time } t = \frac{\sum_{i\geq 3} X_i(t)}{\sum_{\forall i} X_i(t)}$$

I calculated the (undiscounted) number of new HIV infections that occur in the entire population over the time horizon, T.

$$\text{New HIV infections} = \int_0^T \sum_{i\leq 2} \sum_{j\geq 3} \lambda_{i,j}(t) X_i(t) dt$$

Total health benefits for the entire population were measured in discounted quality-adjusted life years (QALYs). I assumed an infinite time horizon to account for health benefits occurring after the intervention duration.

$$\text{QALYs} = \int_0^\infty e^{-rt} \sum_{\forall i} q_i X_i(t)dt$$

Total discounted costs for the entire population were calculated as the sum of annual healthcare costs for all individuals, including costs of antiretroviral treatment, and total screening and counseling costs over the intervention's duration.

$$\begin{aligned}
&\text{Costs} \\
= \; & \int_0^\infty e^{-rt} \sum_{\forall i} c_i X_i(t)dt + \int_0^T e^{-rt} \left[\left(e_{NAAT} c_{NAAT} \right) \psi_{ACUTE} + (c_{ASSAY} + c_{coun}) \psi_{ASSAY} \right] X_1(t)dt \\
+ \; & \int_0^T e^{-rt} \left[(e_{NAAT} c_{NAAT} + c_{WB} + c_{coun} + c_{viral}) sens_{NAAT} \left(\frac{\omega_{ASSAY} - \omega_{NAAT}}{1/\theta_{ACUTE}} \right) \psi_{ACUTE} \right] X_3(t)dt \\
& + \int_0^T e^{-rt} \left[(c_{ASSAY} + c_{WB} + c_{coun}) \left(1 - \frac{\omega_{ASSAY}}{1/\theta_{ACUTE}} \right) \psi_{ASSAY} \right] X_3(t)dt \\
& + \int_0^T e^{-rt} \left[(c_{ASSAY} + c_{WB} + c_{coun})(\psi_{ASSAY} + \psi_5) \right] X_5(t)dt \\
& + \int_0^T e^{-rt} \left[(c_{ASSAY} + c_{WB} + c_{coun})(\psi_{ASSAY} + \psi_7) \right] X_7(t)dt \\
& + \int_0^T e^{-rt} \left[(c_{ASSAY} + c_{WB} + c_{coun})(\psi_{ASSAY} + \psi_{10}) \right] X_{10}(t)dt
\end{aligned}$$

Finally, I calculated the incremental cost-effectiveness ratio (ICER) of each HIV screening strategy, relative to the status quo.

$$\text{ICER} = \frac{Cost_{Intervention} - Cost_{StatusQuo}}{QALY_{Intervention} - QALY_{StatusQuo}}$$

I also calculated the ICER of one screening strategy relative to another, if appropriate.

Supporting Information References

[1] Long EF, Brandeau ML, Owens DK. The cost-effectiveness and population outcomes of expanded HIV screening and antiretroviral treatment in the United States. *Ann Intern Med.* 2010;153:778–789.

[2] Long EF, Brandeau ML, Owens DK. Potential population health outcomes and expenditures of HIV vaccination strategies in the United States. *Vaccine.* 2009;27:5402–5410.

[3] Centers for Disease Control and Prevention (CDC) . Estimates of New HIV Infections in the United States. 2008.

[4] Joint United Nations Programme on HIV/AIDS (UNAIDS) . 2008 Report on the Global AIDS Epidemic. 2008.

[5] Friedman SR, Tempalski B, Cooper H, et al. Estimating numbers of injecting drug users in metropolitan areas for structural analyses of community vulnerability and for assessing relative degrees of service provision for injecting drug users. *J Urban Health.* 2004;81:377–400.

[6] Evans JL, Hahn JA, Page-Shafer K, et al. Gender differences in sexual and injection risk behavior among active young injection drug users in San Francisco (the UFO Study). *J Urban Health.* 2003;80:137–146.

[7] CensusScope . United States Age Distribution. 2000. http://www.censusscope.org/us/chart_age.html.

[8] Centers for Disease Control and Prevention (CDC) . HIV and AIDS among Gay and Bisexual Men. 2010.

[9] Rietmeijer CA, Wolitski RJ, Fishbein M, Corby NH, Cohn DL. Sex hustling, injection drug use, and non-gay identification by men who have sex with men. Associations with high-risk sexual behaviors and condom use. *Sex Transm Dis.* 1998;25:353–360.

[10] Arias E. United States Life Tables, 2001. *Natl Vital Stat Rep.* 2004;52:1-38.

[11] Zaric GS, Barnett PG, Brandeau ML. HIV transmission and the cost-effectiveness of methadone maintenance. *Am J Public Health.* 2000;90:1100-1111.

[12] Fryback DG, Dasbach EJ, Klein R, et al. The Beaver Dam Health Outcomes Study: initial catalog of health-state quality factors. *Med Decis Making.* 1993;13:89–102.

[13] Coco A. The cost-effectiveness of expanded testing for primary HIV infection. *Ann Fam Med.* 2005;3:391–399.

[14] Honiden S, Sundaram V, Nease RF, et al. The effect of diagnosis with HIV infection on health-related quality of life. *Qual Life Res.* 2006;15:69–82.

[15] Schackman BR, Goldie SJ, Freedberg KA, Losina E, Brazier J, Weinstein MC. Comparison of health state utilities using community and patient preference weights derived from a survey of patients with HIV/AIDS. *Med Decis Making.* 2002;22:27–38.

[16] Tengs TO, Lin TH. A meta-analysis of utility estimates for HIV/AIDS. *Med Decis Making.* 2002;22:475–481.

[17] Holtgrave DR, Pinkerton SD. Updates of cost of illness and quality of life estimates for use in economic evaluations of HIV prevention programs. *J Acquir Immune Defic Syndr Hum Retrovirol.* 1997;16:54–62.

[18] Long EF, Brandeau ML, Galvin CM, et al. Effectiveness and cost-effectiveness of strategies to expand antiretroviral therapy in St. Petersburg, Russia. *AIDS.* 2006;20:2207–2215.

[19] Pinkerton SD. How many sexually-acquired HIV infections in the USA are due to acute-phase HIV transmission? *AIDS.* 2007;21:1625–1629.

[20] Patel P, Mackellar D, Simmons P, et al. Detecting acute human immunodeficiency virus infection using 3 different screening immunoassays and nucleic acid amplification testing for human immunodeficiency virus RNA, 2006-2008. *Arch Intern Med.* 2010;170:66–74.

[21] Hollingsworth TD, Anderson RM, Fraser C. HIV-1 transmission, by stage of infection. *J Infect Dis.* 2008;198:687–693.

[22] Sanders GD, Bayoumi AM, Sundaram V, et al. Cost-effectiveness of screening for HIV in the era of highly active antiretroviral therapy. *N Engl J Med.* 2005;352:570-585.

[23] Centers for Disease Control and Prevention (CDC) . Persons tested for HIV–United States, 2006. *MMWR Morb Mortal Wkly Rep.* 2008;57:845–849.

[24] Fiebig EW, Wright DJ, Rawal BD, et al. Dynamics of HIV viremia and antibody seroconversion in plasma donors: implications for diagnosis and staging of primary HIV infection. *AIDS.* 2003;17:1871–1879.

[25] Hecht FM, Busch MP, Rawal B, et al. Use of laboratory tests and clinical symptoms for identification of primary HIV infection. *AIDS.* 2002;16:1119–1129.

[26] Eshleman SH, Khaki L, Laeyendecker O, et al. Detection of individuals with acute HIV-1 infection using the ARCHITECT HIV Ag/Ab Combo assay. *J Acquir Immune Defic Syndr.* 2009;52:121–124.

[27] Patel P, Klausner JD, Bacon OM, et al. Detection of acute HIV infections in high-risk patients in California. *J Acquir Immune Defic Syndr.* 2006;42:75–79.

[28] Westreich DJ, Hudgens MG, Fiscus SA, Pilcher CD. Optimizing screening for acute human immunodeficiency virus infection with pooled nucleic acid amplification tests. *J Clin Microbiol.* 2008;46:1785–1792.

[29] Hutchinson AB, Patel P, Sansom SL, et al. Cost-effectiveness of pooled nucleic acid amplification testing for acute HIV infection after third-generation HIV antibody screening

and rapid testing in the United States: a comparison of three public health settings. *PLoS Med.* 2010;7:e1000342.

[30] Marks G, Crepaz N, Senterfitt JW, Janssen RS. Meta-analysis of high-risk sexual behavior in persons aware and unaware they are infected with HIV in the United States: implications for HIV prevention programs. *J Acquir Immune Defic Syndr.* 2005;39:446–453.

[31] Fox J, White PJ, Macdonald N, et al. Reductions in HIV transmission risk behaviour following diagnosis of primary HIV infection: a cohort of high-risk men who have sex with men. *HIV Med.* 2009;10:432–438.

[32] Steward WT, Remien RH, Higgins JA, et al. Behavior change following diagnosis with acute/early HIV infection-a move to serosorting with other HIV-infected individuals. The NIMH Multisite Acute HIV Infection Study: III. *AIDS Behav.* 2009;13:1054–1060.

[33] Teshale EH, Kamimoto L, Harris N, Li J, Wang H, McKenna MT. Estimated number of HIV-infected persons eligible for and receiving HIV antiretroviral therapy, 2003. 12th Conference on Retroviruses and Opportunistic Infections. 2005. Abstract 167.

[34] National Institute of Mental Health (NIMH) Multisite HIV Prevention Trial Group . The NIMH Multisite HIV Prevention Trial: reducing HIV sexual risk behavior. *Science.* 1998;280:1889–94.

[35] Kamb ML, Fishbein M, Douglas JM, et al. Efficacy of risk-reduction counseling to prevent human immunodeficiency virus and sexually transmitted diseases: a randomized controlled trial. Project RESPECT Study Group. *JAMA.* 1998;280:1161–1167.

[36] Quinn TC, Wawer MJ, Sewankambo N, et al. Viral load and heterosexual transmission of human immunodeficiency virus type 1. Rakai Project Study Group. *N Engl J Med.* 2000;342:921–929.

[37] Abbas UL, Anderson RM, Mellors JW. Potential impact of antiretroviral therapy on HIV-1 transmission and AIDS mortality in resource-limited settings. *J Acquir Immune Defic Syndr.* 2006;41:632–641.

[38] McCormick AW, Walensky RP, Lipsitch M, et al. The effect of antiretroviral therapy on secondary transmission of HIV among men who have sex with men. *Clin Infect Dis.* 2007;44:1115–1122.

[39] Cohen MS, Gay C, Kashuba ADM., Blower S, Paxton L. Narrative review: antiretroviral therapy to prevent the sexual transmission of HIV-1. *Ann Intern Med.* 2007;146:591–601.

[40] Wilson DP, Law MG, Grulich AE, Cooper DA, Kaldor JM. Relation between HIV viral load and infectiousness: a model-based analysis. *Lancet.* 2008;372:314–320.

[41] Vernazza P, Hirschel B, Bernasconi E, Flepp M. HIV transmission under highly active antiretroviral therapy. *Lancet.* 2008;372:1806–1807.

[42] O'Donnell H. The United States Circumcision Century. 2001. http://www.boystoo.com/history/statistics.htm.

[43] Auvert B, Taljaard D, Lagarde E, Sobngwi-Tambekou J, Sitta R, Puren A. Randomized, controlled intervention trial of male circumcision for reduction of HIV infection risk: the ANRS 1265 Trial. *PLoS Med.* 2005;2:e298.

[44] Desai K, Boily M-C, Garnett GP, Masse BR, Moses S, Bailey RC. The role of sexually transmitted infections in male circumcision effectiveness against HIV - insights from clinical trial simulation. *Emerg Themes Epidemiol.* 2006;3:19.

[45] Taylor A, Hutchinson S, Lingappa J, et al. Severe illness and death among injecting drug users in Scotland: a case-control study. *Epidemiol Infect.* 2005;133:193–204.

[46] Kral AH, Lorvick J, Ciccarone D, et al. HIV prevalence and risk behaviors among men who have sex with men and inject drugs in San Francisco. *J Urban Health.* 2005;82:43–50.

[47] Spittal PM, Craib KJP, Wood E, et al. Risk factors for elevated HIV incidence rates among female injection drug users in Vancouver. *CMAJ.* 2002;166:894–899.

[48] Harris ZK. Efficient allocation of resources to prevent HIV infection among injection drug users: the Prevention Point Philadelphia (PPP) needle exchange program. *Health Econ.* 2006;15:147–158.

[49] Prabhu VS, Hutchinson AB, Farnham PG, Sansom SL. Sexually acquired HIV infections in the United States due to acute-phase HIV transmission: an update. *AIDS.* 2009;23:1792–1794.

[50] Wawer MJ, Gray RH, Sewankambo NK, et al. Rates of HIV-1 transmission per coital act, by stage of HIV-1 infection, in Rakai, Uganda. *J Infect Dis.* 2005;191:1403–1409.

[51] Padian NS, Shiboski SC, Glass SO, Vittinghoff E. Heterosexual transmission of human immunodeficiency virus (HIV) in northern California: results from a ten-year study. *Am J Epidemiol.* 1997;146:350–357.

[52] Downs AM, De Vincenzi I. Probability of heterosexual transmission of HIV: relationship to the number of unprotected sexual contacts. European Study Group in Heterosexual Transmission of HIV. *J Acquir Immune Defic Syndr Hum Retrovirol.* 1996;11:388–395.

[53] Mastro TD, Vincenzi I. Probabilities of sexual HIV-1 transmission. *AIDS.* 1996;10 Suppl A:75–82.

[54] Nicolosi A, Correa Leite ML, Musicco M, Arici C, Gavazzeni G, Lazzarin A. The efficiency of male-to-female and female-to-male sexual transmission of the human immun-

odeficiency virus: a study of 730 stable couples. Italian Study Group on HIV Heterosexual Transmission. *Epidemiology.* 1994;5:570–575.

[55] Kaplan EH. Modeling HIV infectivity: must sex acts be counted? *J Acquir Immune Defic Syndr.* 1990;3:55–61.

[56] Jacquez JA, Koopman JS, Simon CP, Longini IM. Role of the primary infection in epidemics of HIV infection in gay cohorts. *J Acquir Immune Defic Syndr.* 1994;7:1169–1184.

[57] Caceres CF, Griensven GJ. Male homosexual transmission of HIV-1. *AIDS.* 1994;8:1051–1061.

[58] Vittinghoff E, Douglas J, Judson F, McKirnan D, MacQueen K, Buchbinder SP. Per-contact risk of human immunodeficiency virus transmission between male sexual partners. *Am J Epidemiol.* 1999;150:306–311.

[59] Pathela P, Hajat A, Schillinger J, Blank S, Sell R, Mostashari F. Discordance between sexual behavior and self-reported sexual identity: a population-based survey of New York City men. *Ann Intern Med.* 2006;145:416–425.

[60] Harawa NT, Greenland S, Bingham TA, et al. Associations of race/ethnicity with HIV prevalence and HIV-related behaviors among young men who have sex with men in 7 urban centers in the United States. *J Acquir Immune Defic Syndr.* 2004;35:526–536.

[61] MacKellar DA, Valleroy LA, Behel S, et al. Unintentional HIV exposures from young men who have sex with men who disclose being HIV-negative. *AIDS.* 2006;20:1637–1644.

[62] Tyndall MW, Patrick D, Spittal P, Li K, O'Shaughnessy MV, Schechter MT. Risky sexual behaviours among injection drugs users with high HIV prevalence: implications for STD control. *Sex Transm Infect.* 2002;78 Suppl 1:170–175.

[63] Bacon O, Lum P, Hahn J, et al. Commercial sex work and risk of HIV infection among young drug-injecting men who have sex with men in San Francisco. *Sex Transm Dis.* 2006;33:228–234.

[64] Brisson M, Boily MC, Masse BR, Adrien A, Leaune V. Highlights of the sexual activity of the heterosexual population in the province of Quebec. *Sex Transm Infect.* 1999;75:296–299.

[65] Fryar CD, Hirsch R, Porter KS, Kottiri B, Brody DJ, Louis T. Drug Use and Sexual Behaviors Reported by Adults: United States, 1999-2002. *Adv Data.* 2007;384:1-14.

[66] Johnson AM, Mercer CH, Erens B, et al. Sexual behaviour in Britain: partnerships, practices, and HIV risk behaviours. *Lancet.* 2001;358:1835–1842.

[67] National Opinion Research Center . General Social Surveys (GSS), 1972-2006 2006. http://sda.berkeley.edu/archive.htm.

[68] Paltiel AD, Weinstein MC, Kimmel AD, et al. Expanded screening for HIV in the United States–an analysis of cost-effectiveness. *N Engl J Med.* 2005;352:586–595.

[69] Pilcher CD, McPherson JT, Leone PA, et al. Real-time, universal screening for acute HIV infection in a routine HIV counseling and testing population. *JAMA.* 2002;288:216–221.

[70] Pilcher CD, Fiscus SA, Nguyen TQ, et al. Detection of acute infections during HIV testing in North Carolina. *N Engl J Med.* 2005;352:1873–1883.

[71] Priddy FH, Pilcher CD, Moore RH, et al. Detection of acute HIV infections in an urban HIV counseling and testing population in the United States. *J Acquir Immune Defic Syndr.* 2007;44:196–202.

[72] Freedberg KA, Losina E, Weinstein MC, et al. The cost effectiveness of combination antiretroviral therapy for HIV disease. *N Engl J Med.* 2001;344:824–831.

[73] Elbeik T, Charlebois E, Nassos P, et al. Quantitative and cost comparison of ultrasensitive human immunodeficiency virus type 1 RNA viral load assays: Bayer bDNA quantiplex versions 3.0 and 2.0 and Roche PCR Amplicor monitor version 1.5. *J Clin Microbiol.* 2000;38:1113–1120.

[74] Bozzette SA, Joyce G, McCaffrey DF, et al. Expenditures for the care of HIV-infected patients in the era of highly active antiretroviral therapy. *N Engl J Med.* 2001;344:817–823.

[75] Schackman BR, Gebo KA, Walensky RP, et al. The lifetime cost of current human immunodeficiency virus care in the United States. *Med Care.* 2006;44:990–997.

[76] Hutchinson AB, Farnham PG, Dean HD, et al. The economic burden of HIV in the United States in the era of highly active antiretroviral therapy: evidence of continuing racial and ethnic differences. *J Acquir Immune Defic Syndr.* 2006;43:451–457.

[77] World Health Organization (WHO) . World Health Statistics. 2007.

[78] Gold MR, Siegel JE, Russell LB, Weinstein MC. *Cost-Effectiveness in Health and Medicine.* New York: Oxford University Press 1996.
