# Supplementary figures and images for: HIV Screening via Fourth-Generation Immunoassay or Nucleic Acid Amplification Test in the United States: A Cost-Effectiveness Analysis
Source: PLoS One. 2011 Nov 16;6(11):e27625. doi: 10.1371/journal.pone.0027625 (PMC3218000; doi:10.1371/journal.pone.0027625)

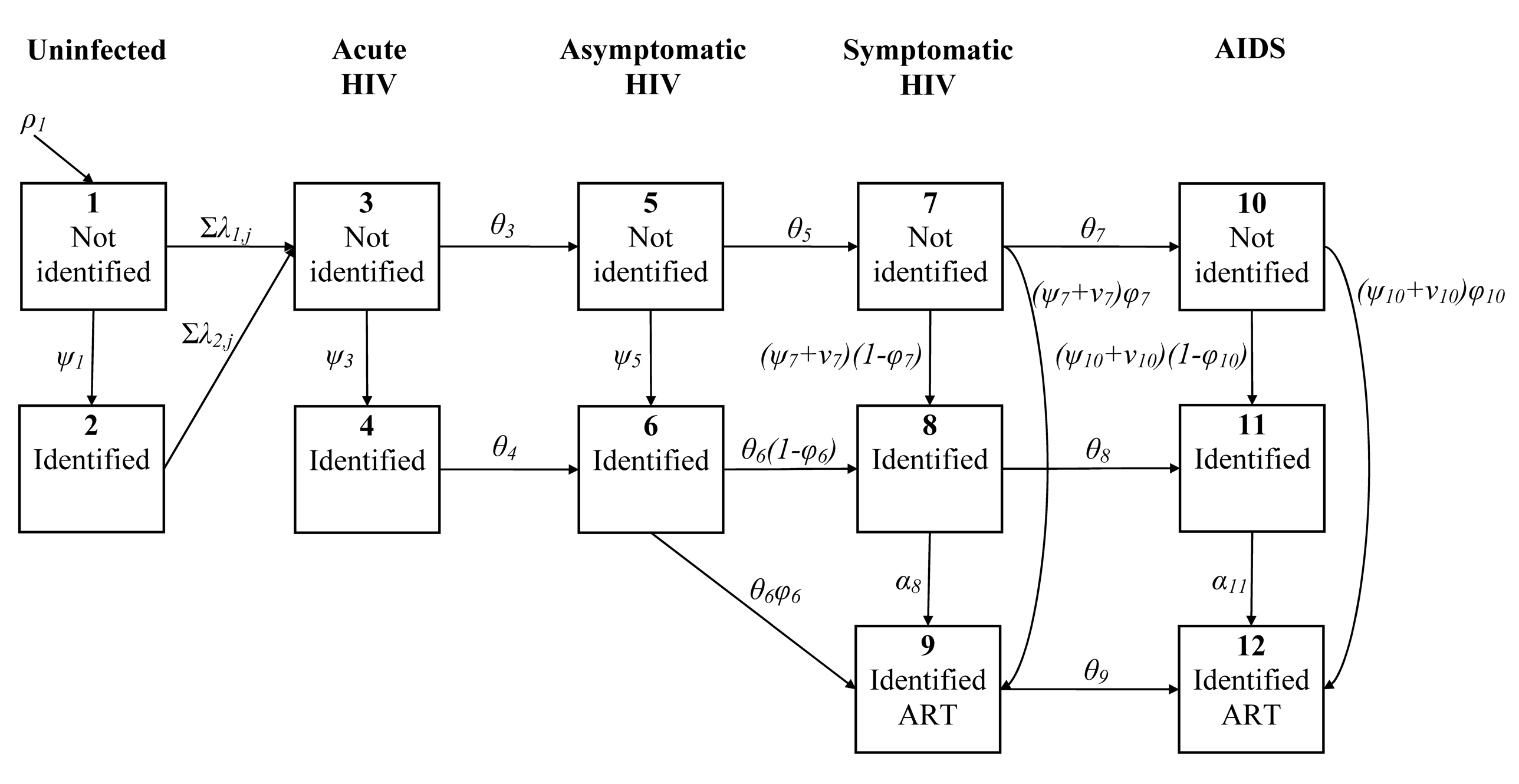

Supplement: Figure S1 — Schematic diagram of HIV transmission model and potential modes of transmission. The boxes represent cohorts of individuals in each disease stage and the arrows represent transitions due to disease transmission, disease progression, mortality, screening, or treatment initiation. ART = antiretroviral therapy. A description of each parameter is given in Table S2. (JPG) [file pone.0027625.s002.jpg]

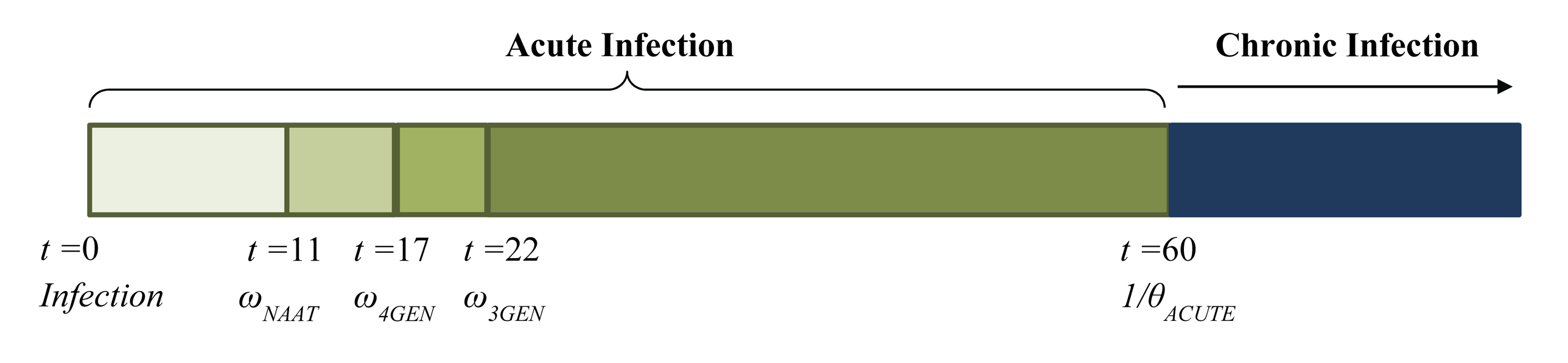

Supplement: Figure S2 — Window period of detection during acute HIV. The figure shows the duration (in days) of the acute infection period (1/θACUTE), and the window period of detection for each test: nucleic acid amplification test (ωNAAT), fourth-generation immunoassay (ω4GEN), and third-generation enzyme linked immunosorbent assay (ω3GEN). (JPG) [file pone.0027625.s003.jpg]
