# Supplementary material for: HIV Screening via Fourth-Generation Immunoassay or Nucleic Acid Amplification Test in the United States: A Cost-Effectiveness Analysis
Source: PLoS One. 2011 Nov 16;6(11):e27625. doi: 10.1371/journal.pone.0027625 (PMC3218000; doi:10.1371/journal.pone.0027625)
Supplement: Table S1 — Modes of HIV transmission. (PDF) [file pone.0027625.s004.pdf]

Table S1: Modes of HIV transmission

| | Male MSM | Male MSM/IDU | Male IDU | Male Other | Female IDU | Female Other |
|---|---|---|---|---|---|---|
| Male MSM | Homosexual | Homosexual | | | Heterosexual | Heterosexual |
| Male MSM/IDU | Homosexual | Homosexual<br>Needle-sharing | Needle-sharing | | Heterosexual<br>Needle-sharing | Heterosexual |
| Male IDU | | Needle-sharing | Needle-sharing | | Heterosexual<br>Needle-sharing | Heterosexual |
| Male Other | | | | | Heterosexual | Heterosexual |
| Female IDU | Heterosexual | Heterosexual<br>Needle-sharing | Heterosexual<br>Needle-sharing | Heterosexual | Needle-sharing | |
| Female Other | Heterosexual | Heterosexual | Heterosexual | Heterosexual | | |

MSM = men who have sex with men; IDU = injection drug user; Other = low-risk general population.
