# Supplementary material for: HIV Screening via Fourth-Generation Immunoassay or Nucleic Acid Amplification Test in the United States: A Cost-Effectiveness Analysis
Source: PLoS One. 2011 Nov 16;6(11):e27625. doi: 10.1371/journal.pone.0027625 (PMC3218000; doi:10.1371/journal.pone.0027625)
Supplement: Table S2 — Summary of key model parameters. (PDF) [file pone.0027625.s005.pdf]

Table S2: Summary of key model parameters

Parameter	Value	Range	Source
**Demographic Parameters**			
Population 15-64 years, $X(0)$			
Male IDU	1,000,000	0.5-1.5 million	[3–7]
Male MSM	4,057,194	3-6 million	[3, 4, 7, 8]
Male IDU/MSM	300,000	200,000-500,000	[3–9]
Male Other	96,022,652	95-100 million	[3, 4, 7]
Female IDU	450,000	300,000-600,000	[3–7]
Female Other	101,632,781	100-105 million	[3, 4, 7]
Initial HIV prevalence, $\sum_{i\geq 3} X_i(0)/\sum_{\forall i} X_i(0)$			
Male IDU	12.9%	10-20%	[3–7]
Male MSM	12.6%	5-20%	[3, 4, 7, 8]
Male IDU/MSM	18.8%	15-30%	[3–9]
Male Other	0.10%	0.05-0.25%	[3, 4, 7]
Female IDU	17.3%	15-30%	[3–7]
Female Other	0.22%	0.10-0.40%	[3, 4, 7]
Annual exit rate, μ_i			
Mortality			
Male	0.0041	0.001-0.005	[10]
Female	0.0024	0.001-0.005	[10]
IDU (excess)	0.025	0-0.05	[11]
Maturation			
Male	0.0111	0.01-0.02	[7]
Female	0.0122	0.01-0.02	[7]
Annual entry rate, ρ_i			
Male	0.0227	0.01-0.05	[7]
Female	0.0213	0.01-0.05	[7]
**Disease Parameters**			
Quality-of-life factor, q_i			
Uninfected	1.0	–	[12]
Acute HIV - Unaware	0.91	0.60-0.95	[13–17]
Acute HIV - Aware	0.89	0.60-0.95	[13–17]
Asymptomatic HIV - Unaware	0.92	0.85-0.95	[14–17]

Parameter	Value	Range	Source
Asymptomatic HIV - Aware	0.89	0.85-0.95	[14–17]
Symptomatic HIV - Unaware	0.79	0.70-0.85	[14–17]
Symptomatic HIV - Aware	0.72	0.70-0.85	[14–17]
Symptomatic HIV - On ART	0.83	0.80-0.87	[14–17]
AIDS - Unaware	0.72	0.60-0.80	[14–17]
AIDS - Aware	0.72	0.60-0.80	[14–17]
AIDS - On ART	0.82	0.80-0.87	[14–17]
IDU (multiplier)	0.90	0.80-1.0	[11, 18]
Annual disease progression rate, θ_i			
Acute HIV	6	4-12	[19–21]
Asymptomatic HIV	0.11	0.10-0.20	[2, 22]
Symptomatic HIV - Untreated	0.33	0.20-0.50	[2, 22]
Symptomatic HIV - On ART	0.06	0.05-0.10	[2, 22]
AIDS - Untreated	0.40	0.20-1.00	[2, 22]
AIDS - On ART	0.25	0.10-0.50	[2, 22]
**HIV Screening Parameters**			
Proportion tested in past 12 months, ψ_i			
High-risk individuals	23%	10-30%	[23]
Low-risk individuals	10%	5-20%	[23]
Symptom-based case finding rate, ν_i			
Symptomatic HIV	10%	0-30%	[22]
AIDS	20%	10-60%	[22]
Window period of detection (days)			
3rd generation assay, ω_{3GEN}	22	14-40	[20, 24, 25]
4th generation assay, ω_{4GEN}	17	10-28	[20, 26]
NAAT test, ω_{NAAT}	11	9-30	[20, 27]
NAAT pooling algorithm sensitivity, $sens_{NAAT}$	95%	90-100%	[28]
NAAT pooling efficiency (tests/specimen), e_{NAAT}	0.11	0.10-1.0	[19, 28]
Proportion tested who receive test results, f_{NAAT}	80%	50-100%	[29]
Reduction in sexual behavior if identified, ϵ_k	50%	20-70%	[30–32]
**Treatment Parameters**			
Fraction starting ART at CD4=350 cells/mm^3, ϕ_i	50%	25-75%	[33]
Annual ART entry rate if CD4<350 cells/mm^3, α_i	0.05	0-0.10	Estimated[22, 33]

Parameter	Value	Range	Source
Reduction in sexual infectivity due to ART, δ_h^s	90%	50-99%	[34–41]
Reduction in injection infectivity due to ART, δ_h^d	50%	25-75%	[18, 22]
**Circumcision Parameters**			
Fraction of males circumcised	70%	50-80%	[42]
Reduction in transmission due to circumcision, δ_c	50%	48-60%	[43, 44]
**Injection Drug Use Parameters**			
Transmission prob. per shared injection, π^k			
Acute HIV	0.016	0.008-0.040	[11, 18, 19, 21, 24]
Asymptomatic HIV	0.002	0.001-0.005	[11, 18]
Symptomatic HIV	0.003	0.001-0.005	[11, 18]
AIDS	0.003	0.001-0.005	[11, 18]
Average injections per year, d_i	200	100-500	[11, 18, 45]
Fraction of injections with shared needles, s_i	20%	10-40%	[11, 46–48]
**Sexual Behavior Parameters**			
Transmission prob. per partnership (F to M), π_{mf}^k			
Acute HIV	0.20	0.10-0.30	[19–21, 24, 49]
Asymptomatic HIV	0.02	0.01-0.04	[21, 36, 37, 50–55]
Symptomatic HIV	0.03	0.01-0.04	[21, 36, 37, 50–55]
AIDS	0.05	0.03-0.06	[21, 36, 37, 50–55]
Transmission prob. per partnership (M to F), π_{fm}^k			
Acute HIV	0.30	0.10-0.40	[19–21, 24, 49]
Asymptomatic HIV	0.03	0.02-0.05	[21, 36, 37, 50–55]
Symptomatic HIV	0.04	0.02-0.05	[21, 36, 37, 50–55]
AIDS	0.06	0.05-0.09	[21, 36, 37, 50–55]
Transmission prob. per partnership (M to M), π_{mm}^k			
Acute HIV	0.40	0.20-0.50	[19–21, 24, 49, 56]
Asymptomatic HIV	0.04	0.02-0.05	[53, 56–58]
Symptomatic HIV	0.05	0.02-0.05	[53, 56–58]
AIDS	0.08	0.06-0.12	[53, 56–58]
Annual number of same-sex partners, n_i^s			
Male MSM	3.0	2.0-5.0	[46, 59–61]
Male IDU/MSM	3.0	2.0-5.0	[46, 60, 61]

Parameter	Value	Range	Source
Condom use with same-sex partners, u_i^s			
Male MSM	40%	30-60%	[9, 46, 59–61]
Male IDU/MSM	40%	30-50%	[46]
Annual number of opposite-sex partners, n_i^o			
Male IDU	3.0	2.0-5.0	[62]
Male MSM	0.1	0-1.0	[59]
Male IDU/MSM	0.1	0-1.0	[63]
Male Other	1.1	0.5-2.0	[59, 64–67]
Female IDU	3.5	2.0-5.0	[62]
Female Other	1.1	0.5-2.0	[64–67]
Condom use with opposite-sex partners, u_i^o			
Male IDU	25%	15-35%	[9, 63]
Male MSM	30%	20-50%	[9, 46]
Male IDU/MSM	30%	20-50%	[46, 63]
Male Other	20%	10-40%	[67]
Female IDU	25%	20-50%	[47, 62]
Female Other	20%	10-40%	[67]
Condom effectiveness, κ	90%	80-100%	[2]
**Cost Parameters**			
Cost of 3rd generation assay, c_{ASSAY}	$15	$10-$25	[13, 22, 27, 68]
Cost of Western Blot confirmatory test, c_{WB}	$40	$25-$50	[22, 68]
Cost of NAAT, c_{NAAT}	$120	$100-$150	[13, 27, 28, 69–71]
Cost of quantitative viral load assay, c_{viral}	$120	$100-$140	[72, 73]
Cost of HIV counseling, c_{coun}	$60	$40-$100	[22, 68]
Annual HIV-related healthcare costs, c_i			
Asymptomatic HIV	$3,967	$3,000-$6,000	[74, 75]
Symptomatic HIV - Untreated	$6,660	$5,000-$9,000	[74, 75]
Symptomatic HIV - On ART	$5,937	$5,000-$7,000	[74, 75]
AIDS - Untreated	$21,000	$15,000-$25,000	[74–76]
AIDS - On ART	$9,557	$6,000-$17,000	[22, 75]
Annual non-HIV-related healthcare costs, c_i	$6,728	$5,000-$8,000	[77]
Annual cost of ART, c_{ART}	$14,974	$12,000-$18,000	[22, 68, 75, 76]
Annual cost of ancillary IDU services, c_i	$2,500	$1,000-$5,000	[11]
Annual discount rate, r	3%	0-5%	[78]
